# Swelling Behavior of Polyacrylamide–Cellulose Nanocrystal Hydrogels: Swelling Kinetics, Temperature, and pH Effects

**DOI:** 10.3390/ma12132080

**Published:** 2019-06-28

**Authors:** Tippabattini Jayaramudu, Hyun-U Ko, Hyun Chan Kim, Jung Woong Kim, Jaehwan Kim

**Affiliations:** 1 Center for Nanocellulose Future Composites, Department of Mechanical Engineering, Inha University, 100 Inha-Ro, Michuhol-Gu, Incheon 22212, Korea; 2 Laboratory of Material Sciences, Instituto de Quimica de Recursos Naturales, Universidad de Talca, Talca 747, Chile

**Keywords:** hydrogels, swelling, cellulose nanocrystal, diffusion, pH-sensitive

## Abstract

This paper reports swelling behavior of cellulose nanocrystal (CNC)-based polyacrylamide hydrogels prepared by a radical polymerization. The CNC acts as a nanofiller through the formation of complexation and intermolecular interaction. FTIR spectroscopy and XRD studies confirmed the formation of intermolecular bonds between the acrylamide and hydroxyl groups of CNC. The swelling ratio and water retention were studied in de-ionized (DI) water at room temperature, and the temperature effect on the swelling ratio was investigated. Further, the pH effect on the swelling ratio was studied with different temperature levels. Increasing the pH with temperature, the prepared hydrogel shows 6 times higher swelling ratio than the initial condition. The swelling kinetics of the developed hydrogels explains that the diffusion mechanism is Fickian diffusion mechanism. Since the developed hydrogels have good swelling behaviors with respect to pH and temperature, they can be used as smart materials in the field of controlled drug delivery applications.

## 1. Introduction

Hydrogels are widely utilized polymers with fascinating properties and predominantly applied in various modern scientific and technological fields [1,2,3,4,5]. Hydrogels have special characteristic features, that is, swelling behavior in water/biological fluids; due to this behavior, hydrogels are broadly used in many fields, including biomedical tissue engineering, drug delivery, sensors, actuators, and metal/dyes adsorption applications [6,7,8,9,10]. Therefore, the swelling behavior plays a significant role in hydrogel technology. The capacity of swelling behavior in hydrogels occurs as a result of ionization, which makes it possible to absorb water arising in hydrophilic functional groups attached to the cross-linked polymer backbone and through the difference in swelling osmatic pressure between the gel phase and solvent phase. Generally, hydrogels are insoluble three-dimensional hydrophilic cross-linked and tissue-like soft polymeric network structures. These network structures are achieved from a cross-linked polymer using various techniques and classified according to their synthesis techniques [5]. However, copolymerization/cross-linking free-radical polymerization techniques are most commonly used to develop hydrogels by reacting hydrophilic monomers with multifunctional cross-linkers [11]. Among the hydrophilic monomers, acrylamide/modified acrylamide is widely used in hydrogel technology due to its hydrophilic functional groups. Because of these functional groups, it is able to swell drastically in the presence of water/biological fluids without solubility. Acrylamide-based hydrogels are widely used in agriculture, tissue engineering, and biomedical and wastewater treatment [12,13]. To date, considerable studies have been done to improve the mechanical, chemical, and electromechanical properties of polyacrylamide hydrogels by blending nanofillers such as polymeric nanoparticles, inorganic clays, and metal nanoparticles [14,15,16]. Recently, cellulose nanocrystals (CNCs) were blended in polyacrylamide to reinforce their elastic, dielectric, and electromechanical properties, which are applicable for soft electroactive materials in an active lens [17].

Cellulose nanocrystals (CNCs) are made from acid hydrolysis of the cellulose. Cellulose is an eco-friendly organic material with low cost and high thermal stability, and it is biodegradable and biocompatible. The structure of cellulose was first demonstrated by Staudinger in 1920 through the process of acetylation and deacetylation, which also identified that anhydroglucose units are covalently bonded and able to make macromolecular chains [18]. Covalent bonds occur between the β-1,4-glycosidic bonds of the C1 and C4 of the β-D-anhydroglucopyranose monomer units and provide linear shape. The chemical stability of the cellulose is estimated based on the hydrolytic attack at the β-1,4-glycosidic linkages between the repeated units. However, cellulose consists of crystalline and amorphous domains, which are together arranged randomly, and the properties of cellulose are mainly dependent on the arrangement of these domains. The amorphous domains are removed via a simple hydrolysis reaction that can effectively deliver CNCs for many advanced industrial applications of cellulose [19,20,21]. In general, CNCs are rod/needle-shaped crystalline material, which is produced by concentrated sulfuric acid hydrolysis of cellulose. Normally, CNCs are less than 300 nm in length and 20 nm in width, depending on the cellulose resources [22]. Since CNC has rich hydroxyl (-OH) groups on its surface, it can be easily blended with other polymers. Owing to these properties, CNCs are broadly used in hydrogel technology to improve their physical and chemical properties for various fields, such as drug delivery, tissue engineering, and biomedical applications [23,24,25]. For example, CNC-reinforced gelatin hydrogels were reported for controlled drug delivery applications, using rice husks as a resource of cellulose [23]. CNC-reinforced alginate hydrogel beads were fabricated for methylene blue adsorption [26]. Recently, CNC-reinforced, mechanically stiff, and bioactive hybrid hydrogels were developed for bone tissue engineering applications [27]. CNC was incorporated into polyvinyl alcohol (PVA) to develop nontoxic and transparent electroactive hydrogels [28]. Lately, polyacrylamide (PAM)–CNC hydrogels was developed through grafting copolymerization and the shear storage modulus, compression strength, and elastic modulus of the system were studied [29]. However, in-depth analysis of its swelling kinetics and pH and temperature effects were not conducted. 

In the present investigation, we firstly developed CNCs through acid hydrolysis of cellulose. Then, polyacrylamide–CNC (PAC) hydrogels were produced via free-radical polymerization. The PAC hydrogels developed in the presence of different concentrations of CNCs using acrylamide monomer, cross-linker, and initiators. The prepared CNC and their composite hydrogels were characterized by atomic force microscopy (AFM), Fourier transform infrared (FTIR) spectroscopy, and X-ray diffraction (XRD) studies. The thermal stability and mechanical properties were studied using thermogravimetric analysis (TGA) and compression studies. Furthermore, we studied their swelling studies in different pH solutions, temperatures, and combinations of pH and temperature effect and swelling kinetics studies. 

## 2. Materials and Methods 

### 2.1. Materials

Cotton pulp (MVE, DPw-4580) was supplied by Buckeye Technology Inc. (Memphis, TN, USA). Acrylamide (Am), N,N1-methylenebisacrylamide (MBA), ammonium per sulfate (APS), N,N,N′,N′-tetramethylrthylenediamine (TMEDA), and sodium hydroxide (NaOH) were purchased from Sigma-Aldrich, Yongin, Gyeonggi-do, Korea. All chemicals were used without further purification. De-ionized (DI) water was used throughout the experimentation. 

### 2.2. Preparation of CNC–PAC Hydrogels

Firstly, CNC suspension was prepared using the acid hydrolysis technique as previously reported [30]. In a brief explanation, 10 g of cotton pulp was dispersed in 64% of sulfuric acid (H_2_SO_4_) under constant magnetic stirring (300 rpm) at 60 °C over a period of 2 h. Later, the hydrolysis reaction was stopped through the addition of 10 times excess cold DI water, followed by successive centrifugation (30 min, 11,000 rpm), and finally, the suspension was dialyzed overnight to attain neutrality pH = 7. The 0.5 wt% of CNC suspension was prepared via re-dispersion of CNC in DI water, stored in a glass vial at 4 °C until use. Before hydrolysis, an alkaline (1 M NaOH) treatment was carried out to remove the noncellulosic components from the cotton pulp to improve the CNC quality.

**Figure 1:**
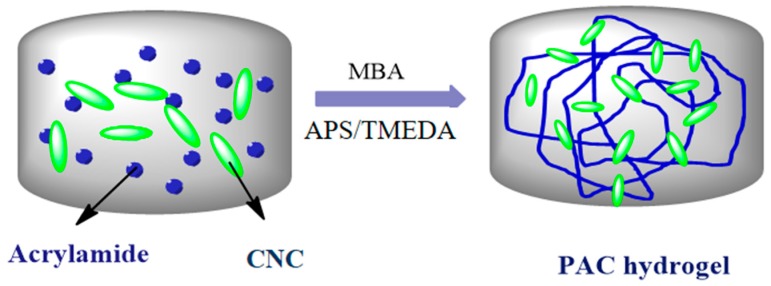
Schematics of polyacrylamide– cellulose nanocrystal (PAC) hydrogel formation.

The PAC hydrogels were prepared using the standard procedure reported previously [2,10]. In brief, 1 wt% of (based on Am concentration) CNC was dispersed in 5 mL of DI water under constant magnetic stirring with 200 rpm. Then, 14.08 mM of Am was dissolved in this solution under the same stirring condition until Am was dissolved. To the solution, MBA and APS/TMEDA were added as per the specifications given in Table 1, and the mixture was stirred for another 30 min. The solution was poured into a petri dish and kept in an oven at 45 °C, where a hydrogel was formed. The formed hydrogel was transferred into a 250 mL beaker containing 100 mL of DI water in order to remove unreacted monomers, cross-linker, and pre-polymer from the hydrogel, and the water was repeatedly changed every 6 h up to 48 h [6]. Similarly, CNC varied (1, 3, and 5 wt. %) PAC hydrogels and the pristine polyacrylamide hydrogel were synthesized using the above procedure, and the developed hydrogels were named as PAm, PAC1, PAC2m and PAC3, respectively, as shown in Table 1. Figure 1 shows the schematics of PAC hydrogel formation.

## 3. Characterization

### 3.1. Physical and Chemical Characteristics

At first, the morphology of the prepared CNC was observed using an AFM (Dimension 3100, Veeco, San Jose, CA, USA) in the tapping mode. The CNC specimen was made by vacuum spraying the prepared CNC on a cleaned silicon wafer. To study the formation of the PAC hydrogels, FTIR spectra were taken. The FTIR samples were prepared by completely drying the prepared hydrogels in the oven at 60 °C for 6 h. The samples were examined on an FTIR spectrometer (Veratex-80, Bruker Optics, Billerica, MA, USA) using the KBr pellet method between 400–4000 cm^−1^. The thermal behavior of the prepared hydrogels was studied via thermogravimetric analysis (TGA) and differential thermal analysis (DTA) (STA 409 PC, Selb, Germany), at a heating rate of 10 °C/min under a constant nitrogen flow (20 mL/min). Wide angle X-ray diffraction was studied using Cu Kα target radiation at 40 kV and 50 mA, at a scanning rate of 0.015°/min. The diffraction angle was varied from 5° to 45°. The surface and cross-section morphologies of the prepared hydrogels were studied using field emission scanning electron microscopy (FE-SEM, S-4000, Hitachi, Tokyo, Japan). The air-dried hydrogels were coated with platinum, and an accelerating voltage of 10 kV was used during imagining. 

### 3.2. Swelling and Water Retention

The swelling ratio was calculated by weighting the dried and wet hydrogels. The completely dried and pre-weighed PAm and PAC hydrogels were equilibrated in 50 mL of DI water in a 100 mL beaker, and the water-observed hydrogels were weighed after removing the surface excess water with a water-sucking filter paper at certain time intervals up to equilibrium using analytical balance (GH-200, A&D weighing, Tokyo, Japan). Then, the equilibrated hydrogels were put in petri dishes at room temperature, and the hydrogels were re-weighted at certain time intervals until they had reached saturated weight values. The swelling ratio (S_g/g_) and % of water retention can be calculated using the following equations:

(1) $$Swelling\;ratio\;\left(S_{g/g}\right)=\frac{W_{t}-W_{o}}{W_{o}},$$

(2) $$Water\;retention\;\left(\%\right)=\frac{W_{t}-W_{o}}{W_{d}-W_{o}},$$

where *W_t_* is the weight of the swollen hydrogel at time intervals, *W_o_* is the weight of dried hydrogels, and *W_d_* is the initial weight of the swollen hydrogel. Moreover, the swelling behavior was analyzed in different pH values, ranging from 2 to 12 solutions.

## 4. Results and Discussion

### 4.1. PAC Hydrogel Formation 

**Figure 2:**
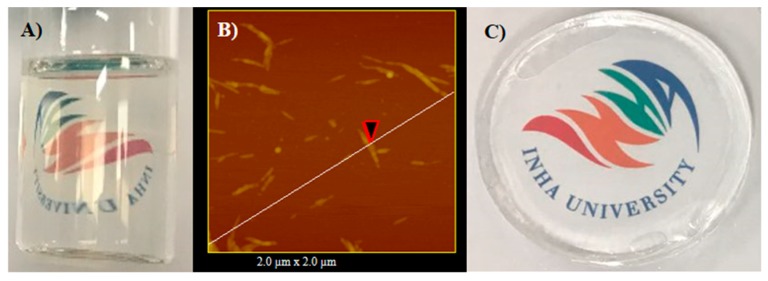
(**A**) Photograph of the prepared cellulose nanocrystal (CNC) suspension, (**B**) atomic force microscopy (AFM) image of the prepared CNCs, and (**C**) photograph of the prepared PAC hydrogel.

**Figure 3:**
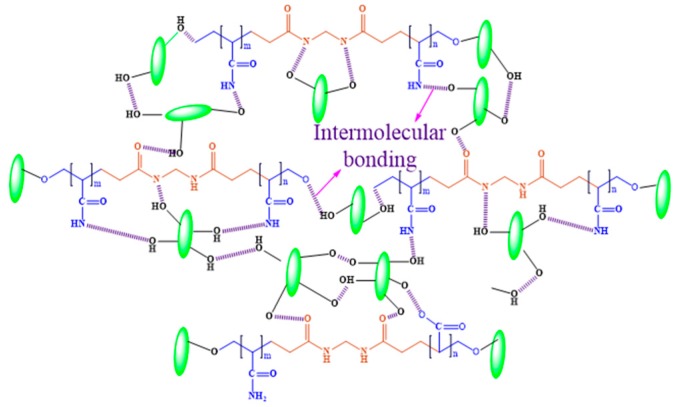
PAC hydrogel formation mechanism.

The PAC hydrogels were prepared through free radical polymerization. Initially, rod shape and transparent CNCs were prepared via acid hydrolysis. Figure 2A shows a photograph of the transparent CNC suspension prepared in this experiment. The morphology of the prepared CNCs was confirmed by AFM, as shown in Figure 2B. CNCs have a rod-like shape with a diameter of 25–40 nm. The transparency of the prepared CNCs is associated with their nano-size. Figure 2C shows the photograph of the prepared hydrogel, PAC3, which shows its transparency. Figure 3 shows a possible formation mechanism of the PAC hydrogel. The Am monomer polymerized in the presence of CNCs and formed the PAC hydrogel. 

**Figure 4:**
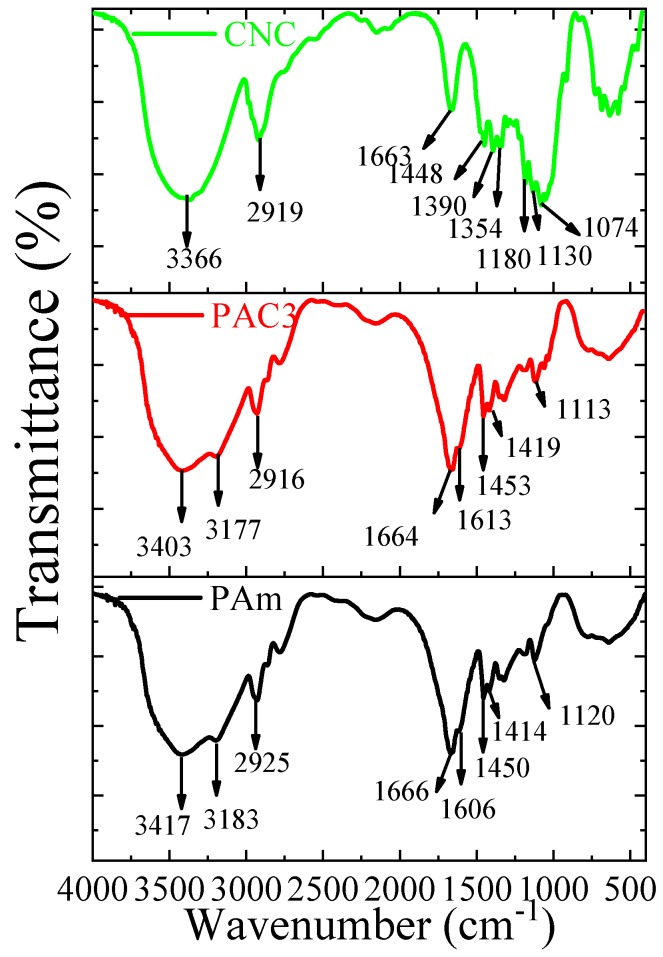
FTIR Spectra of PAm, CNC, and PAC3.

The prepared PAC hydrogels and their chemical interactions were confirmed by FTIR spectra. Figure 4 shows FTIR spectra of the CNC, Pam, and PAC hydrogels. The CNC shows a broad peak at 3366 cm^−1^, which are related to the O–H stretching vibration. The C–H stretching vibration peak is shown at 2919 cm^−1^. Peaks at 1663 cm^−1^ and 1448 cm^−1^ were related to the O–H bending and CH_2_ scissoring motion in cellulose. Characteristics peaks were at 1390 cm^−1^ (C–H bending), 1354 cm^−1^ (O–H in plane bending), 1337 cm^−1^ (CH_2_ wagging), 1180 cm^−1^ (C–C ring stretching), 1130 cm^−1^ (C–O–C glycosidic ether), 1074 cm^−1^ (C-O-C pyranose ring stretching), and 835 cm^−1^ (glycosidic linkages) [27,31,32,33]. The PAm hydrogel shows characteristic peaks at 3417 cm^−1^ and 3183 cm^−1^ related to the stretching vibration of amide functional group (NH_2_) and 2925 cm^−1^ stretching vibrations CH_2_. Peaks at 1666 cm^−1^ and 1606 cm^−1^ are owing to the C=O stretching and NH_2_ bending vibrations, respectively. Peaks at 1450 cm^−1^, 1414 cm^−1^, and 1120 cm^−1^ are related to the –CH_2_ scissoring and CN and N–H stretching vibrations, whereas the PAC3 hydrogel shows all the above characteristic peaks of the PAm, with slight shifts in their peaks (3403, 3177, 2916, 1664, 1613, 1453, 1419, and 1113 cm^−1^), which might be associated with the presence of CNC. This result indicates that CNC was well interacted with PAm through the formation of intermolecular bonds between the acrylamide and hydroxyl groups of CNC. During the formation, the hydroxyl groups of CNCs form a physical cross-linked structure with polyacrylamide chains, which leads to the formation of PAC hydrogel.

### 4.2. Physical Characteristics

**Figure 5:**
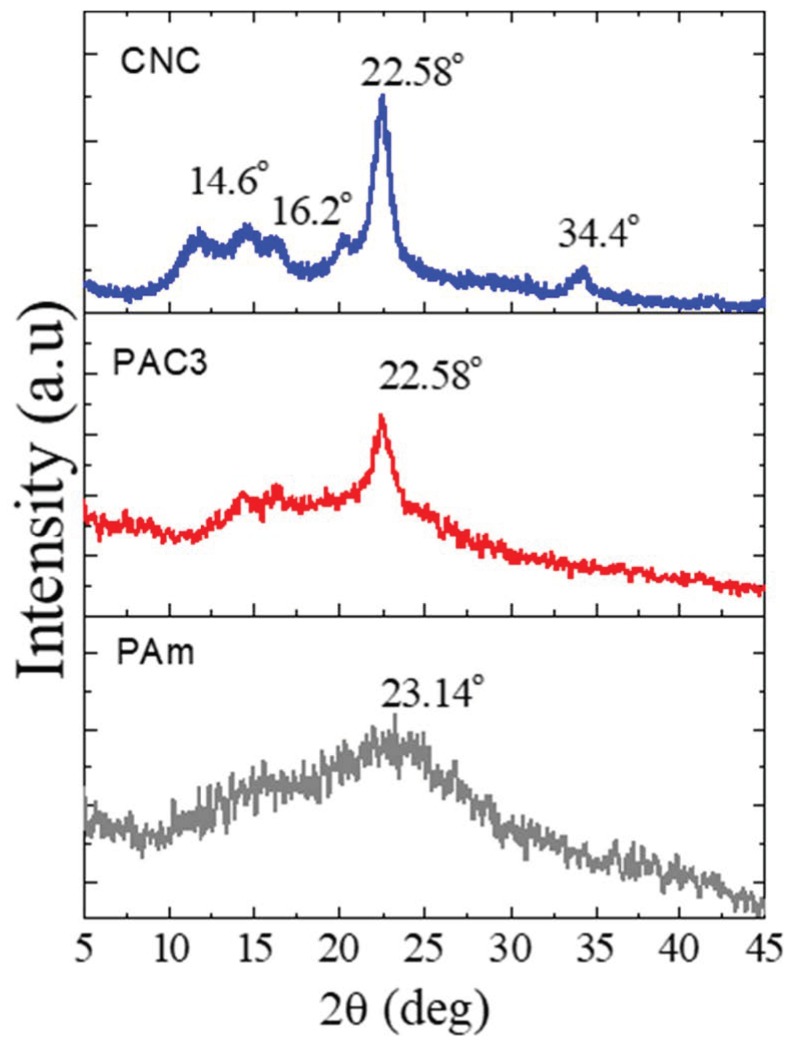
XRD spectra of PAm, CNC, and PAC3.

Figure 5 shows the XRD patterns of the CNC, Pam, and PAC3. It is an important analysis to understand the nature of the hydrogels developed. CNC displays four well-defined crystalline diffraction peaks at 2θ = 14.6°, 16.2°, 22.58°, and 34.4°, respectively [20]. PAm hydrogel exhibits a broad diffraction peak at 2θ = 23.2°, indicating an amorphous nature of the hydrogel. After blending CNC in polyacrylamide, PAC3 shows some crystalline characteristic peaks of CNC, 22.58° and 16.2° in an XRD pattern; meanwhile, the broad characteristic peak of PAm appears noticeably. This indicates that CNCs are dispersed in PAm without losing their crystalline characteristics. Recently, similar results were reported, and the intensity of the peak increased with the CNC content in CNC-based hybrid hydrogels [27].

**Figure 6:**
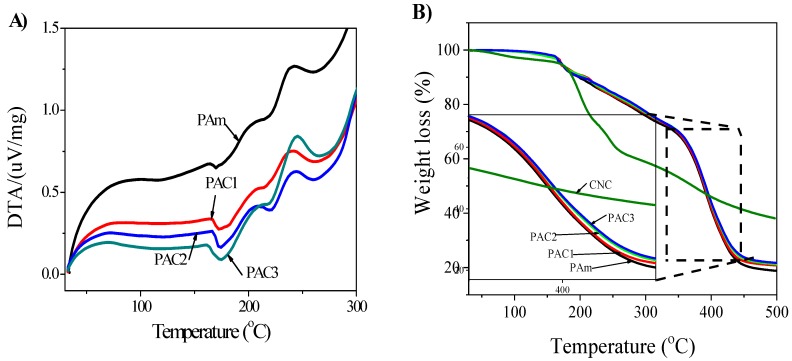
(**A**) Differential thermal analysis (DTA) analysis of PAm and PAC hydrogels; (**B**) TGA analysis of CNC, Pam, and PAC hydrogels.

Figure 6 shows the DTA and TGA analysis results of the prepared hydrogels. DTA and TGA are useful techniques to characterize polymeric materials. The analysis results explain the glass transition (Tg) temperature and thermal stability of the polymeric materials. In Figure 6A, the PAm hydrogels show endothermic peak at 169.6 °C, and this peak is slightly shifted by the addition of CNC (PAC1 = 172.5 °C). The endothermic peak further shifts up as the CNC content increases in the PAC hydrogels (PAC3 = 174.8 °C), which is associated with the formation of intermolecular bonds between two materials. Similarly, the thermal stability was also affected by the addition of CNC and the thermal stability as shown in Figure 6B. A 2% weight loss was observed in all the samples at below 100 °C due to the removal of absorbed water molecules, except CNC. In the prepared PAC hydrogels, three illustrious thermal degradation stages are shown within the following temperature ranges: 34–163 °C for the evaporation of water molecules, 163–338 °C for the degradation of functional groups, and 338–451 °C for the polymer backbone decomposition. Note that different weight residuals are observed at 500 °C: 18.75, 20.48, 21.18, and 22.01 wt. % for PAm, PAC1, PAC2, and PAC3, respectively. Overall, the TGA results indicate that PAm has lower thermal stability than PAC hydrogels, and the thermal stability of the PAC hydrogels increases along the CNC content.

**Figure 7:**
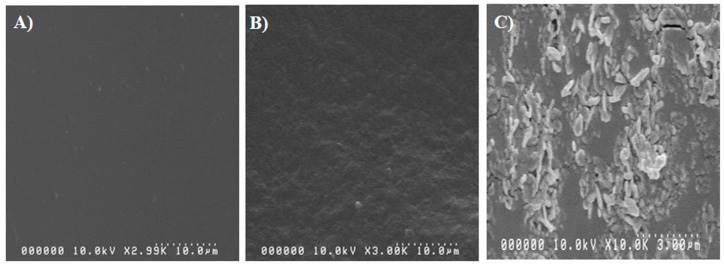
SEM images of (**A**) surface of PAm, (**B**) surface of PAC3, and (**C**) cross-section of PAC3.

The surface morphology of the hydrogels was confirmed by SEM. Figure 7 shows the SEM images of the prepared hydrogels. The SEM observation reveals that PAm exhibits a smooth surface area (Figure 7A), whereas PAC3 shows a rough surface area caused by the CNC distributed in the hydrogel matrix (Figure 7B). Furthermore, a cross-sectional SEM image of PAC3 clearly shows the presence of CNC in the polyacrylamide matrix (Figure 7C). This indicates that most of the CNCs are dispersed in the hydrogel matrix through intermolecular hydrogen bonds between polyacrylamide and CNCs. This result was confirmed by the previous FTIR and XRD studies. However, some aggregations of CNCs are shown. Note that above PAC3, high CNC concentration hydrogels are not easy to prepare because they do not have hydrogel formation, and severe aggregation of CNC occurs.

### 4.3. Swelling and Water Retention

**Figure 8:**
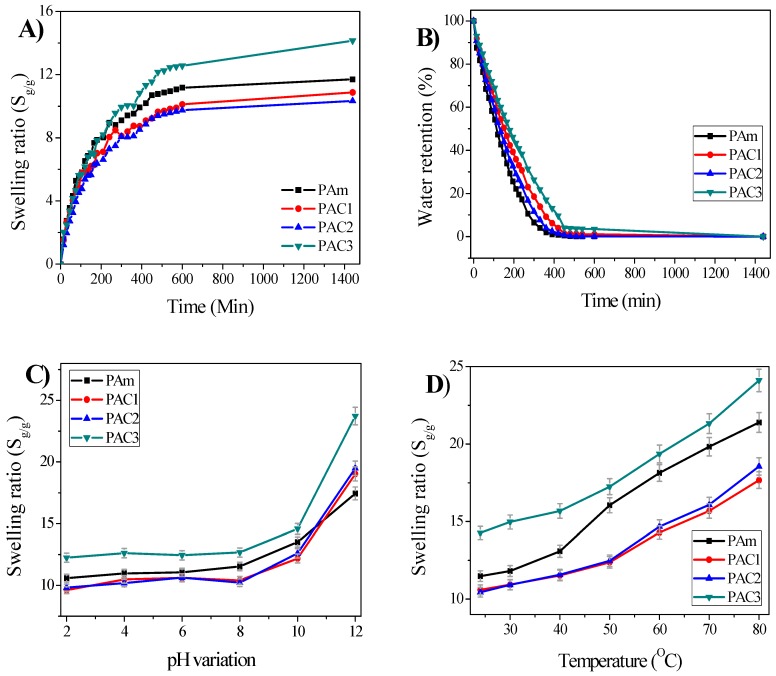
(**A**) Swelling ratio studies in de-ionized (DI) water, (**B**) water retention studies of PAm and PAC hydrogels, (**C**) swelling ratio at different pHs (2–12), and (**D**) temperature effect of swelling ratio in DI water.

Swelling is one of the most important parameters in hydrogel technology. The effect of CNC concentration on the prepared hydrogels was studied via swelling and de-swelling tests. The swelling ratio was measured as a function of time and CNC content. Figure 8A shows the swelling ratios of the prepared hydrogels. The swelling ratios gradually increase with the swelling time at room temperature. Note that the CNC content affects the swelling ratios. For example, PAm shows S_g/g_ = 11.69%, which decreases in the case of PAC1 (10.87%) and PAC2 (10.33%). This might be due to the increase of physical cross-linking and reduced porosity in the hydrogels. However, PAC3 shows a higher swelling ratio than PAm, S_g/g_ = 14.14%, because of remnant CNCs that are shown in Figure 7C as aggregates. Beyond the swelling, the equilibrated hydrogels were removed from the DI water and de-swelled at room temperature for 24 h, which is exactly the reverse of the swelling process, known as water retention. Figure 8B shows the water retention of the prepared hydrogels. Water retention capacity gradually decreases with the time, depending on CNC content. PAm loses absorbed water quickly and evaporates approximately 90% of water content within 5.5 h. Interestingly, the water retention capacity of PAC3 increases from 5.5 to 7.5 h, owing to the hydrogen bonding formation between the water molecules and functional groups of the hydrogel matrix. The enhanced bonding formation delays the evaporation of the absorbed water molecules so as to improve water retention capacity. In a recent study, a similar phenomenon was reported in rice husk ash-based superabsorbent hydrogels [30]. Overall, the addition of CNC improved the swelling ratio and water retention capacity of PAC hydrogels.

The effect of pH on the swelling behavior of the prepared hydrogels was investigated at room temperature for 24 h. Various pH solutions were prepared and adjusted using 0.1 M HCl/0.1 M NaOH solutions with a pH meter (Orion Star A211 pH Benchtop Meter, Thermo Scientific, Beverly, MA, USA). The swelling ratio of the hydrogels at different pH values was calculated using Equation (1), and the results are shown in Figure 8C. The swelling ratio is lower at below pH = 2, and increasing the pH, the swelling ratio slightly increases up to pH = 6. When the pH increases from 8 to 12, the swelling ratio drastically increases. In the literature, several studies explained the swelling ratio behavior at different pH levels [30,31,33,34]. Mostly, at lower pH levels, the H^+^ ions are higher, and these ions form additional physical cross-linking with the functional groups of the hydrogel network. Owing to the formation of this physical cross-linking, the hydrogel network tends to shrink, and the swelling ratio decreases at lower pH levels. Further increasing the pH decreases the formation of the additional physical cross-linking so as to decrease the H^+^ ions and increase the OH– ions with increasing the pH. In general, CNCs have a large number of –OH groups in their structure [35]. It is noticeable that the swelling ratio increases at a high pH, which is attributed to the repulsion of pH solution and the hydrogel network. Recently, a similar phenomenon was reported in the study of carclazyte-based carboxymethyl cellulose-g-poly(acrylic acid-co-acrylamide) superabsorbent hydrogels [36]. However, within the range of pH 6−8, the swelling ratio is almost the same, which is attributed to the neutrality of the pH solutions.

The effect of temperature on the swelling ratio of the prepared hydrogels was investigated. The results are shown in Figure 8D. The prepared hydrogels were swollen at different temperatures from room temperature to 80 °C, and the swelling ratio was calculated when the time was 1400 min. Increasing the temperature, the swelling ratio was consequently enhanced. Generally, hydrogels swell in water through the expansion of their chain networks due to the interaction between the polymeric chain networks and water molecules, which occurs through the entanglement of the interpenetrated (such as capillary, osmotic, and hydration forces) polymeric hydrogel network. When the temperature increases, the interaction behavior disturbs the disentanglement of interpenetrated polymeric chains and destruction of hydrogen bonding between polymer molecules occurs. Thus, the swelling ratio increases [37]. 

**Figure 9:**
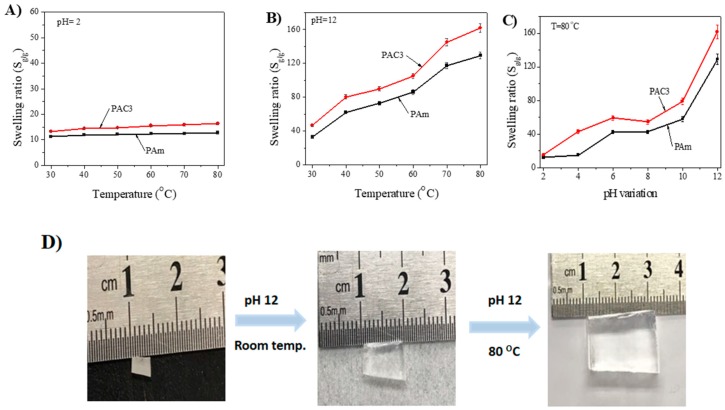
Swelling ratio studies (**A**), pH 2 (**B**) pH 12 at various temperatures, (**C**) swelling ratio at 80 °C in different pH solutions, and (**D**) digital images of swollen hydrogels in pH 12 room temperature and 80 °C.

Based on the pH swelling study and temperature effect on the swelling ratio, the effect of temperature on the pH swelling ratio was studied at lower and higher pH values at different temperatures. Figure 9A,B shows the temperature effect on pH = 2 and pH = 12 swelling ratios. In both pH values, the swelling ratio increases with the temperature increase: When the temperature changes from 30 to 80 °C, the swelling ratio of PAC3 increases from 13.2 to 16.3 for pH = 2 and 46.3 g to 161.7 for pH = 12, respectively. As anticipated, PAm hydrogel shows a smaller swelling ratio than PAC3. Figure 9C shows the different pH swelling ratio values at 80 °C. Similarly, the values increase with increasing the pH level. Overall, PAC3 shows a 4 times higher swelling ratio at pH = 12 than pH = 2. Note that pH and temperature affect the swelling ratio simultaneously. For better understanding, digital images of the dried hydrogel, room temperature, and 80 °C swollen hydrogel at pH = 12 are shown in Figure 9D.

### 4.4. Swelling Kinetics

**Figure 10:**
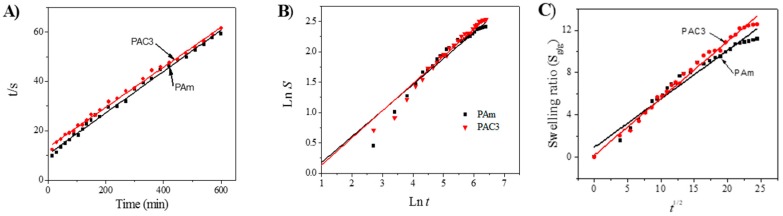
Swelling kinetics curves of PAm and PAC3 in DI water: (**A**) *t/s* verses *t* curves, (**B**) *Ln S* verses *Ln t*, and (**C**) swelling ratio verses *t^1/2^* curves.

The swelling property plays an important role in hydrogel technology, and various techniques are available to study their swelling kinetics mechanism in the literature [38,39,40,41]. Among them, a simple kinetic analysis equation is used in a second order equation:

(3) $$\frac{ds}{dt}=k_{s}{\left(S_{eq}-S\right)}^{2}$$

where *ds/dt*, *k_s_*, *S_eq_*, and *S* refer to the rehydration ratio, swelling rate constant, equilibrium swelling, and swelling ratio at *t*, respectively. After definite integration by applying the initial condition such as *S* = 0 at *t* = 0 and *S* = *S_eq_*, Equation (3) converts to:

(4) $$\frac{t}{S}=A+Bt$$

where *A = 1/k_s_S_eq_^2^* is the initial swelling rate of the hydrogel composite and *k_s_* is the swelling rate constant, and *B(=1/S_eq_)* is the converse of the maximum or equilibrium swelling. In order to investigate the above kinetic model for the PAC hydrogels, the graph was drawn t/s versus t in Figure 10A, and the slopes and intersections of plotted lines give the initial rate of swelling (*r_i_*), the swelling rate constant (*k_s_*), and the theoretical equilibrium (*S_eq_*) values, shown in Table 2. According to the results, the theoretical swelling ratio is close to the experimental swelling ratios. The swelling mechanism of the hydrogels can be determined using the below equation:

(5) $$swelling\;ratio\;\left(S\right)=\left(\frac{W_{s}-W_{d}}{W_{d}}\right)=kt^{n}$$

where *S*, *W_s_*, and *W_d_* are the fraction swelling ratio at time *t*, the weight of the swollen hydrogels at time *t*, and the weight of the dried hydrogels at time *t* = 0, respectively, *k* is the swelling constant and *n* is the swelling exponent, which indicates the water transport mechanism [42,43]. The above equation was used to estimate the *n* value using up to 60% of the swelling ratio values, then the plot was drawn between the *ln S* versus *ln t* and slope of the obtain straight lines gives the swelling exponent value *n*. Based on the *n* values, the diffusion mechanism is identified. When *n* ≤ 0.5, the diffusion mechanism is a Fickian diffusion, and when 0.5 < *n* < 1.0, it indicates non-Fickian diffusion or anomalous diffusion. In anomalous diffusion, the diffusion and relaxation are said to be isochronally effective. If the *n* value is exactly equal to 1, the diffusion mechanism is designated as Case II diffusion, and when the *n* value is above 1 (*n* > 1) it is called a super Case II diffusion mechanism, which is very rarely possible [44]. Figure 10B shows the slope and intercept of the straight lines that can give *n* and *k* values, shown in Table 2. The developed hydrogels show a Fickian type diffusion mechanism, which is more suitable for biomedical applications, especially in controlled drug delivery applications. The swelling constant *k* can be described with diffusion coefficient *D* for a Fickian diffusion. As *n* is approximately taken as 0.5 for an ideal Fickian diffusion, the swelling ratio of the hydrogels can be written using the equation:

(6) $$S=\sqrt{\frac{D}{\pi r^{2}}}\sqrt{t},$$

where *D*, *r*, *S*, and *t* represent the diffusion coefficient of the hydrogel, the radius of the hydrogel, swelling ratio, and time, respectively. $\sqrt{Dt}$ represents a diffusion length. The diffusion coefficient value *D* was calculated by drawing the plot *S* versus *t^1/2^* (Figure 10C), and the slope of the line gives the diffusion coefficient value *D*. The diffusion coefficient value of PAm hydrogel is 0.4577 cm^2^·s^−1^, while the case of PAC3 is 0.5377 cm^2^·s^−1^, which indicates a faster diffusion rate of molecules in PAC3 than PAm. A large diffusion coefficient is directly associated with the swelling ratio. The higher water diffusion might be associated with the presence of CNC in the hydrogel network. Further, as the CNC concentration increased, the water retention capacity of the prepared hydrogels was improved. The kinetic parameters obtained are shown in Table 2.

Hydrogels that can produce large swelling with pH and temperature are attractive for biomedical applications such as drug delivery, active wound healing, and adaptive scafolds for artificial organs. Softness and shape change behaviors are advantages of these materials. However, biocompatibility, mitigation of toxicity, and reliability are challenges that should be overcome.

## 5. Conclusions

The PAC hydrogels were prepared via a radical polymerization in an aqueous solution of CNCs using monomers, cross-linkers, and initiators. CNC acted as a nanofiller through the formation of intermolecular interaction. The formation of intermolecular bonds between the acrylamide and hydroxyl groups of CNC in the prepared hydrogels were confirmed by FTIR and XRD studies. DTA analysis results explained the endothermic peak increase as CNC content increased. Similarly, the thermal stability was improved by the addition of CNCs in the synthesized hydrogels, and SEM studies revealed the CNCs well dispersed in the hydrogels.

The swelling ratio gradually increased with time at room temperature, and the CNC content affected the swelling ratio. The PAC hydrogels showed excellent swelling behaviors in terms of pH, temperature, and temperature with pH. Increasing the pH with temperature, the PAC3 hydrogel exhibited a 6 times higher swelling ratio than the initial condition. A swelling kinetics study explained that the diffusion mechanism of PAC hydrogels is a Fickian diffusion mechanism. The resulting PAC hydrogels are interesting as potential biomaterials due to their good swelling (pH and temperature) and can be used in the field of biomedical implants, especially in controlled drug delivery applications.

## Figures and Tables

**Table 1:** Feed composition of PAC hydrogels.

Hydrogel Code	AM (mM)	0.5 wt. % CNC (Wt. %)	MBA (mM)	APS (mM)	TMEDA (mM)	Swelling Ratio S_g/g_
PAm	14.08	0	0.648	2.191	0.8605	11.69355
PAC1	14.08	1	0.648	2.191	0.8605	10.8675
PAC2	14.08	3	0.648	2.191	0.8605	10.3317
PAC3	14.08	5	0.648	2.191	0.8605	14.1400

**Table 2:** Swelling kinetics parameters of PAC hydrogels.

Hydrogel Code	Swelling Exponent (*n*)	Diffusion Coefficient (*D*) cm^2^·s^−1^	Initial Swelling Rate (*r_i_*) [g water/g hydrogel]/min	Theoretical Equilibrium Swelling (*S_eq_*) [g Water/ g Hydrogel]	Swelling Rate Constant (*k_s_*) [g Hydrogel/g Water/min]
PAm	0.4279	0.4577	0.0744	10.53	8.9158
PAC3	0.4473	0.5377	0.0619	13.184	14.1123
